# The intergroup dynamics of political cynicism: how perceived discrimination, outsiderness, and social capital relate to political cynicism among Moroccan and Turkish Belgians

**DOI:** 10.3389/fsoc.2024.1437835

**Published:** 2024-08-29

**Authors:** Koen Abts, Jef Van Den Abbeele, Cecil Meeusen, Bart Meuleman

**Affiliations:** Center for Sociological Research, Faculty of Social Sciences, KU Leuven, Leuven, Belgium

**Keywords:** political cynicism, established ethnic minorities, perceived discrimination, outsiderness, social capital

## Abstract

**Introduction:**

This study examines how intergroup dynamics shape political cynicism among Belgians of Turkish and Moroccan descent. Concretely, we examine whether perceptions of discrimination, feelings of ethnic outsiderness and social capital (in terms of associational membership) can explain minorities’ belief that political elites are selfish, incompetent, and immoral.

**Methods:**

We analyse data from the Belgian Ethnic Minorities Election Study 2014.

**Results:**

Arguing that political cynicism includes blame attribution towards the political establishment, we distinguish between perceived group discrimination by the government, on the labor market, and in everyday life. As expected, political cynicism is closely related to perceptions of government discrimination, with no observed correlation with discrimination in the other domains. Next, we show that perceived ethnic outsiderness is also strongly related to increased feelings of political cynicism, further reinforcing the argument that cynics are concerned with their ethnic group’s excluded status and position in society. Finally, associational membership is only related to lower political cynicism when it is generated exclusively within ethnic boundaries; there was no relationship with cross-ethnic social capital.

**Discussion:**

Our findings show that intergroup indicators are highly relevant for understanding minorities’ political cynicism, but that the intergroup dynamics operate in complex and nuanced ways.

## 1 Introduction

Political cynicism can pose a significant threat to the legitimacy of democracy. While high levels of political trust are often considered as vital for democratic systems, fostering law-abiding citizens and public participation (Dalton, 2004; Marien and Hooghe, 2011), a certain level of political distrust can serve as a catalyst for responsiveness to citizen demands (Norris, 1999; Rosanvallon, 2008). Whereas the latter might be true for political distrust or skepticism, many scholars agree that political cynicism can jeopardize the very essence of democracy (Cappella and Jamieson, 1997; Rijkhoff, 2018). Political cynicism goes beyond distrust, in the sense that cynical citizens perceive the political arena as characterized by incompetence, dishonesty, self-interest and immorality (Krouwel and Abts, 2007; Rijkhoff, 2015). Political cynics are often portrayed as apolitical, referring to a retreat from politics, or even a ‘toxic form of anti-political paralysis’ (Stanley, 2007: 386). Cynics seem to be utterly convinced that the entire political system is corrupt and that political elites are immoral and incompetent, making their preconceptions more resistant to change than distrusting or skeptic citizens (Eisinger, 2000; Krouwel and Abts, 2007; Stanley, 2007).

This study delves into the realm of political cynicism within a very particular group: established ethnic minorities living in Western Europe. Given that political cynicism plays a pivotal role in shaping minority groups’ perceptions of the inclusivity and responsiveness of democratic systems (Otjes and Krouwel, 2018), it is reasonable to argue that ethnic minorities in Western Europe may harbor valid reasons for their cynicism. Ethnic minorities often face discrimination and are confronted with a majority society in which a significant part of the population remains reluctant to fully accept them as equal members of the civil society (Alanya et al., 2017; Auspurg et al., 2017). Such negative experiences are known to be associated with negative, and even alienated, attitudes towards the host society (Döring, 2007; Tolsma et al., 2012; Verkuyten, 2016) and might particularly resonate with a disillusioned and cynical worldview. The political inclusion of ethnic minorities is an essential component of democratic legitimacy. Consequently, there is a need to improve our understanding of how political cynicism functions within this specific group, characterized by unique intergroup experiences.

We investigate three explanatory mechanisms of political cynicism that are relevant to ethnic minority groups: perceived discrimination, feelings of outsiderness, and social capital (measured as associational membership). First, we assess whether different types of perceived discrimination drive political cynicism. On the one hand, we disentangle individual- and group-level discrimination, arguing that the cynic’s negative views of political elites are more likely linked to an awareness of the ethnic group’s systemic disadvantage and discrimination. On the other hand, we differentiate between perceptions of group-level discrimination in various domains and hypothesize that political cynicism is more closely tied to perceived group discrimination by government agencies than to instances of group-level discrimination experienced in daily life or the labor market. This distinction stems from the fact that political cynicism involves assigning blame specifically to the political establishment. Second, this study explores the fatalistic feeling of ethnic outsiderness – the sense of being excluded as a full member of civil society, regardless of one’s efforts and with no prospects for improvement – as a potential driver of political cynicism. By underscoring a lack of belief in emancipation, feelings of outsiderness can resonate strongly with a cynical worldview. One way of coping with discrimination and exclusion is through active engagement in the ethnic community. In Europe, many ethnic minorities engage in dense networks of co-ethnic and cross-ethnic organizations that provide access to different types of social capital (Fennema and Tillie, 1999; Jacobs et al., 2004). However, critics of multicultural society argue that co-ethnic social engagement is inward looking and can lead to a ‘minority culture of mistrust’ (Kumlin and Rothstein, 2007: 2). Other scholars, however, find a positive correlation between co-ethnic civic participation and democratic engagement (Togeby, 2004; Giugni et al., 2014). We examine whether co-ethnic social capital is differentially related to political cynicism compared to cross-ethnic social capital.

To test our hypotheses, we use data from the Belgian Ethnic Minorities Election Study 2014 (Swyngedouw et al., 2016), a probability-based survey specifically designed to target Moroccan and Turkish minorities in two Belgian cities (Antwerp and Liège). The Turkish and Moroccan communities in Belgium represent two relatively large immigrant groups that play a prominent role in shaping Belgium’s multicultural landscape. Despite being well-established communities with multiple generations living in the country, both groups still face challenges related to social inclusion, education and employment.

## 2 Defining political cynicism

Although closely related, political cynicism cannot simply be equated with a lack of trust in political elites. Both theoretically and empirically, mere distrust is not sufficient to label someone as cynical (Krouwel and Abts, 2007). Instead, political cynicism constitutes a state of mind characterized by the belief that politicians are driven solely by self-interest (Dekker, 2006). In the eyes of the cynic it is a truism that political elites are selfish, dishonest, immoral, and incompetent, and that the entire political system is corrupt (Krouwel and Abts, 2007). Political cynicism is therefore understood as a condition marked by a loss of faith in political ideals and values, in which expressions of suspicion and contempt play an important role (Dekker, 2006; Stanley, 2007). While political trust is an evaluation of whether or not political authorities and institutions adhere to normative expectations (Miller and Listhaug, 1990), political cynicism involves a profound, antagonistic distrust of political entities that are perceived to be guided by self-interest and to act completely out of touch with citizens (Rijkhoff, 2015).

In the literature, cynicism is usually also distinguished from political skepticism and alienation (Finifter, 1970; Abts, 2006; Dekker, 2006; Krouwel and Abts, 2007). Skeptical citizens doubt whether to trust or distrust but are still democratically committed and have a relatively open mind towards politics. Their skepticism forces them to constantly monitor the political leaders in order to make up their minds or even change them. Cynics, on the other hand, have already made up their minds. Political cynicism resonates with negative prejudice towards political elites and feelings of disillusionment and hopelessness, making it more resistant to change than distrust or skepticism (Eisinger, 2000; Krouwel and Abts, 2007; Stanley, 2007). However, despite their withdrawal from politics, cynics are not necessarily completely alienated from democratic society. While political alienation involves a rejection of democratic ideals and norms (Finifter, 1970), cynicism is often defined as a perceived discrepancy between democratic ideals and the political reality.

Political cynicism also overlaps with, but is distinct from, the concept of (external) political efficacy, i.e., the extent to which citizens have confidence in the responsiveness of the political system to their concerns and believe that their involvement can meaningfully influence political decisions. While the target of political efficacy is the individual (the self’s ability to change politics), the referent of cynicism is the political domain (politicians, political institutions, and the political system as a whole) (Rijkhoff, 2015).

## 3 Intergroup inequalities: perceived discrimination, outsiderness and political cynicism

To understand the relationship between perceived discrimination and political cynicism, the distinction – introduced by Runciman (1966) – between ‘egoistic relative deprivation’ (i.e., the feeling of being treated unfairly as an individual) and ‘fraternalistic relative deprivation’ (i.e., the feeling of being treated unfairly as a group, regardless of one’s own experiences) and its differential consequences for political protest is instructive. Personally experienced discrimination is generally associated with personal enhancement strategies and low self-esteem (Runciman, 1966; Verkuyten and Nekuee, 1999; Bourguignon Seron et al., 2006), whereas perceived group discrimination primarily affects minorities’ political attitudes and behavior (Sanders et al., 2014; Galle et al., 2019). Concerning political cynicism, perceived group discrimination seems to be particularly relevant, as it refers to a sense of group position and perceived unequal intergroup relations (Blumer, 1958). Cynics’ prejudice against political elites may be partly a result of the awareness of their group’s status as a structurally deprived and discriminated group. Through a sense of group position, minorities may understand that their disadvantaged status is structurally embedded in a political system that perpetuates inequality (Avery, 2009), which translates into blame attribution to the political establishment, which is perceived as fundamentally dishonest and driven by self-interest.

Whereas most research on political cynicism among stigmatized minority groups concerns racial minorities (Miller et al., 1981; Avery, 2006, 2009; Mangum, 2016), they illustrate that cynical feelings originate from a shared understanding of illegitimate inequalities between one’s own group and the dominant group in terms of recognition, redistribution and power. In Western Europe, ethnic minority groups are often victims of discrimination, especially those with an Islamic background (Alanya et al., 2017; Auspurg et al., 2017). Because of this stigmatized status, they may understand their disadvantaged status as a product of a morally corrupt political system. *We therefore expect that perceived group discrimination is positively related to political cynicism (H1)*, whereas we do not expect personally experiences discrimination to be related to political cynicism.

In our study, individual-level discrimination is measured in a general and non-specific manner, with respondents being asked whether they have personally experienced some discrimination due to their descent in the past 5 years. Group-level discrimination, on the other hand, is explicitly measured across three different domains: interactions with the government, the labor market, and in daily life. The attitudinal consequences of discrimination are generally targeted at who is considered to be the agent of unfair treatment (Monteith and Spicer, 2000). Since discrimination can vary considerably across life domains (Maxwell, 2015), domain-specific experiences may have different attitudinal consequences (Meeusen et al., 2019). Following Alanya et al. (2017), we distinguish between discrimination in everyday life, on the labor market, and by government agencies. In particular, perceived group discrimination for which the government can be held responsible seems to be related to political disengagement among ethnic minorities (Sanders et al., 2014). As political cynicism involves blame attribution and prejudice towards political objects, it is more likely to be related to perceived discrimination by the government, as it may stimulate minorities to associate their disadvantaged status with the policies of a morally corrupt and discriminatory political establishment. In the case of perceived group discrimination on the labor market or in everyday life, responsibility is not attributed to politics, as non-governmental others (e.g., employers and majority group citizens) are to blame. Also, perceived group discrimination by government agencies resonates particularly with the disillusionment that characterizes the mind of the cynic, because it highlights the politicized and institutionalized nature of inequality and the mismatch between democratic ideals (of equal treatment) and the political reality. This is arguably an important justification for disengagement from politics (Stanley, 2007). Since perceived discrimination on the labor market or in everyday life does not represent a particular view on the political status quo, we do not expect a direct relationship with political cynicism. In sum, we hypothesize that *perceived group discrimination by government agencies (H2) is related to higher levels of political cynicism*. We do not expect a relationship between perceived group discrimination on the labor market and perceived group discrimination in everyday life and political cynicism.

In addition to perceived discrimination, political cynicism among ethnic minorities may be related to another perception of intergroup inequality, namely perceived ethnic outsiderness - namely the feeling that one’s group is not a full part of society (Mehta, 2017). This concept refers to feelings of permanent exclusion within the broader social fabric, and feelings of not being fully respected as capable citizens simply because of one’s group status. Even more than perceptions of discrimination, ethnic outsiderness emphasizes the structural and unchangeable position of the ethnic minority group as established outsiders. The awareness that one’s own ethnic group is (and always will be) an outsider in society confronts the subject with established groups who are not concerned with the ethnic group’s fate in society. Whereas perceived discrimination refers to past experiences, outsiderness captures fatalism about the future group position. This concept has not been considered in research on political cynicism yet.

When people are convinced that their ethnic group is not considered to be part of society, they tend to develop a sense of being left behind by the established insiders, primarily embodied by the political elites. Ethnic outsiderness re-emphasizes the distinctive but unaccepted social and cultural characteristics of the ethnic minority group compared with the dominant majority. Such a divide might lead minority group members to feel less politically represented, empowered and efficacious (Abrajano and Alvarez, 2010). According to this argument, people who do not feel represented and respected as full citizens may not only lose faith in political authorities, but may also judge them as dishonest, immoral and superior (cf. Marschall and Shah, 2007; Wolak, 2017; Koch, 2019). Therefore, in addition to perceived group discrimination, we expect that *higher levels of perceived ethnic outsiderness are related to higher levels of political cynicism (H3).*

## 4 Associational membership and political cynicism

One strategy to address discrimination and ethnic exclusion is to leverage resources from social network connections. In contexts characterized by discrimination and exclusion, participation in civic organizations provides ethnic minorities with social support that might otherwise be inaccessible (Maliepaard et al., 2015). This social capital can spill over into trust in democratic institutions by fostering habits of cooperation and public-spiritedness, which may reduce political cynicism (Putnam, 2000). Although the causal relationship is debated, studies generally suggest that individuals who are involved in civic organizations tend to exhibit lower levels of political cynicism (Schyns and Nuus, 2008). Particularly, organizations characterized by ‘bridging’ social capital—based on weak ties across social divides—are considered beneficial for democracy, as opposed to ‘bonding’ social capital, which relies on strong ties within a specific group (Stolle and Rochon, 1998; Berger et al., 2004). Bonding capital, found in co-ethnic organizations, can sometimes reinforce outgroup suspicion and hinder full integration (Kumlin and Rothstein, 2007).

Empirical research typically shows that co-ethnic membership does not lead to political disengagement. On the contrary, bonding social capital often positively impacts political engagement and satisfaction (Fennema and Tillie, 1999; Heath et al., 2013). Members of co-ethnic organizations are more politically active, though this may affect informal rather than formal political mobilization (Togeby, 2004; Giugni et al., 2014). Therefore, these studies do not support the notion that co-ethnic social capital negatively impacts political engagement and trust, thereby countering critics’ concerns that multiculturalist policies may lead to segregation and decreased social cohesion (Putnam, 2007). Instead, co-ethnic organizations can empower ethnic minorities and enhance their sense of political efficacy (Cappella and Jamieson, 1997; Fennema and Tillie, 1999; Fleischmann et al., 2016). Therefore, we expect that both *co-ethnic (H4a) and cross-ethnic (H4b) membership to be negatively related to political cynicism.*

## 5 The case of Moroccan and Turkish minorities in Belgium

To test these hypotheses, this study examines the attitudes of members of the Turkish and Moroccan communities in Belgium, whose presence in the country originated from Belgium’s active immigration policy aimed at recruiting temporary migrant workers for the coal mines in the 1960s. After the oil crisis in the 1970s, the Belgian government decided to stop the active recruitment of unskilled workers. However, mainly through family reunification and marriage migration, Turks and Moroccans continued to migrate to Belgium, further consolidating the permanent presence of these communities in Belgian society. Today, the Turkish and Moroccan communities are still among the largest national groups coming from non-EU countries (Statbel, 2021; Jacques et al., 2022). In terms of foreign descent, the Moroccan community is the largest ethnic minority group in Belgium in 2020, followed by the Italian, French, Dutch and Turkish communities (Statbel, 2021).

Despite the majority having obtained Belgian citizenship (Gsir et al., 2015; Statbel, 2021), Moroccan and Turkish minorities often occupy disadvantaged socio-economic positions. Unemployment rates are three to four times higher among Belgians of Moroccan and Turkish descent (9.7 and 8.1% respectively, compared to 2.4% for Belgians with native-born parents), and those who are employed are two times more likely to be in the lowest daily wage bracket (0-€100/day). Regarding civic participation, there is only a small difference in membership of organizations between majority Belgians and the Turkish and Moroccan communities. However, the latter have a clear preference for religious organizations and trade unions over socio-cultural associations (Jacques et al., 2022).

In our study, we focus on two major cities in Belgium: Antwerp and Liège. Antwerp, the largest city in the Dutch-speaking part of Belgium (Flanders), and Liège, the largest city in the French-speaking part of Belgium (Wallonia), were selected due to their substantial Turkish and Moroccan populations. Antwerp, a major port city with a significant industrial history, has been a key destination for Turkish and Moroccan labor migrants since the early 1970s. The Turkish and Moroccan communities in Antwerp constitute approximately 15% of the city’s total population. Liège also has a notable presence of Turkish and Moroccan migrants (10%), although the demographic and socio-economic context differs from that of Antwerp. By including both cities, our study aims to capture a diverse range of experiences within the Turkish and Moroccan communities across Belgium’s linguistic regions.

## 6 Materials and methods

### 6.1 Data

We use data from the Belgian Ethnic Minorities Election Study 2014 (BEMES) (Swyngedouw et al., 2016), which includes first-and second-generation Belgian citizens of Moroccan and Turkish descent aged 18 and over. Ethnic background was not defined by self-categorization, but by means of information on the place of birth of the respondent’s parents from municipal population registers. A total of 878 completed surveys were collected (50.9% female, 50.9% Turkish). All respondents were randomly selected from the population registers of Liège (located in the French-speaking part of Belgium, Wallonia) and Antwerp (located in the Dutch-speaking part of Belgium, Flanders) and were interviewed using computer-assisted personal interviewing (CAPI), with a response rate of 34.9%. Post-stratification weight coefficients based on age, gender, city of residence, and ethnic background (Moroccan vs. Turkish) were applied.

### 6.2 Measurements

#### 6.2.1 Dependent variable

Political cynicism is measured with four Likert-type items (5-point agree-disagree items). As political cynicism does not necessarily target all levels or entities of government, we followed the advice by Rijkhoff (2015) to address different political objects and components of cynicism. Two items capture attitudes towards political parties and refer to beliefs that political parties are opportunistic and out of touch with voters’ views: ‘It makes no sense to vote, the parties do what they want to do anyway’ and ‘Parties are only interested in my vote, not in my opinion’. The two other items probe attitudes towards politicians and refer to beliefs that politicians are incompetent, dishonest and self-satisfied: ‘Most politicians promise a lot, but do not do anything’ and ‘Once elected, politicians think they are better than people like me’. By means of Confirmatory Factor Analysis (CFA), the measurement quality and dimensionality of this instrument is established. A one-factor model including an error covariance between the two indicators measuring cynicism towards politicians yields a very good fit (*χ*^2^ = 2.071; df = 1; RMSEA = 0.035; CFI = 0.999; TLI = 0.994; see Table 1). All factor loadings are above 0.60, indicating that the scale measures political cynicism in a sufficiently reliable and valid way. In addition, using a multigroup CFA model, we tested for measurement equivalence of the three factors of group discrimination between respondents of Turkish and Moroccan descent. This analysis shows that scalar invariance is present (see Supplementary Table A1), indicating that the measurements are comparable across groups and that both groups interpret the items in a very similar manner.

**Table 1:** CFA results for political cynicism.

	Political cynicism	
	% Agree + Completely agree	Standardized factor loadings
There’s no sense in voting; the parties do what they want to do anyway.	52.8	0.718
Parties are only interested in my vote, not in my opinion.	73.2	0.771
Most politicians promise a lot, but do not do anything.	70.4	0.689
As soon as they are elected, politicians think they are better than people like me.	63.6	0.602

Model fit: Chi-square = 2.071; df = 1; RMSEA = 0.35; CFI = 0.999; TLI = 0.994.

#### 6.2.2 Independent variables

Perceived group discrimination is measured by three latent factors, each referring to a different domain. Perceived group discrimination by the government is measured with four 5-point Likert items referring to differential treatment by government agencies in general and by the local social assistance agency. Perceived group discrimination in the labor market is measured with three 5-point Likert items about perceived inequalities on the job market. Perceived group discrimination in everyday life is measured by asking respondents how often people of their group experience hostility or discrimination because of their descent at school, at the workplace, when going out, on the street, or on public transport. The fit of a one-factor model, pooling perceptions of the different types of discrimination was unsatisfactory (*χ*^2^ = 946.321; df = 35; RMSEA = 0.173; CFI = 0.598; TLI = 0.483). A three-factor measurement model of perceived group discrimination fits the data adequately (*χ*^2^ = 102.112; df = 32; RMSEA = 0.050; CFI = 0.969; TLI = 0.956; see Supplementary Table A2). The correlations between the three latent factors of perceived group discrimination range from 0.141 to 0.645, indicating that they are related, but sufficiently distinguishable.

Personally experienced discrimination is measured on a 5-point scale and coded as a dummy variable, distinguishing two groups: respondents who have personally experienced at least some discrimination because of their descent in the last 5 years and respondents who have not personally experienced any discrimination. Perceived ethnic outsiderness was measured with the following question: ‘*Some people say that a person of Turkish/Moroccan descent can never fully participate in Belgian society, no matter how hard they try. Others say that a person of Turkish/Moroccan descent can participate fully in Belgian society. Which of these two opinions do you agree with?*’ Respondents agreeing with the first statement are respondents who perceive ethnic outsiderness.

To measure membership of associations, respondents were given a list of association types, and asked about their membership using the following question: ‘*Could you tell me if you are an active member of one of the following organizations. By active member we mean that you have participated in one or more activities organized by the association in the last year.*’ The associations mentioned were the following: (1) Community center; (2) Sports club; (3) School committee or parents’ committee; (4) A recreational club like youth, theater, music or literature; (5) A religious, mosque or church association; (6) A neighborhood committee; (7) An anti-racism organization; (8) Another organization. If respondents answered ‘yes’ to a particular type of organization, a follow-up question was asked: ‘*So you are member of a [include organization type]. How many of the members of [organization type] are of the same descent as you? Most of them – About half – Some of them*’ If the response is ‘Most of them’ we consider the membership as co-ethnic; otherwise as cross-ethnic. Based on all membership questions, 4 categories are constructed: (1) No membership: respondents who have no active memberships; (2) Cross-ethnic only: All the respondents’ memberships are cross-ethnic; (3) Co-ethnic only: All the respondents’ memberships are co-ethnic; (4) Both cross-ethnic and co-ethnic: the respondent has cross-ethnic as well as co-ethnic memberships.

*Control* *variables* – Several other variables may affect political cynicism and confound its relationship with perceived discrimination, perceived ethnic outsiderness, and association membership (Maxwell, 2010; Röder and Mühlau, 2012; Michelson, 2016). Educational attainment is coded into three categories based on the highest degree attained: ‘lower secondary’, ‘higher secondary’, and ‘tertiary’. Subjective income is operationalized by asking respondents which of the following situations best describes their financial situation: ‘enough income and able to save money’; ‘just enough income to make ends meet’; ‘not enough income and regularly have troubles to make ends meet’. Occupational status is coded in two categories based on the Erikson-Goldthorpe-Portocarero class categories (Ganzeboom and Treiman, 1996): ‘manual worker’ (EGP category ≥7) and ‘non-manual worker’. A more complex operationalization of occupational class (including a separate class for students) did not significantly affect the effect sizes.

To operationalize religious practice, we distinguish between respondents who reported they are not Muslim, non–strictly practicing Muslims, and strictly practicing Muslims. A respondent was categorized as a strictly practicing Muslim if he/she reported having always fasted during the last Ramadan and praying at least five times a day. The category ‘other’ refers to a small and heterogeneous group of non-Muslims. We decided not to include mosque attendance in the operationalization, as this leads to a strong gender bias for strictly practicing Muslims. Generational status is coded in three categories: ‘first generation’ (not born in Belgium), ‘1.5-generation’ (not born in Belgium but migrated before the age of 15), and ‘second generation’ (born in Belgium). Age (in years) is included as a metric scale. City of residence is coded as a dummy variable with respondents living in Antwerp as the reference category. Descriptive statistics for all variables presented in Supplementary Table A3.

### 6.3 Methods

To test the hypotheses, we use a structural equation modeling (SEM) approach performed in Lavaan version 0.6–7 (Rosseel, 2012). In order to gain insight into the interplay between the different sets of predictors, we estimate four sequential models. In the baseline model, we estimate the relationship between the control variables and political cynicism. Next, we build upon the baseline model by including perceived individual and group discrimination as well as perceived ethnic outsiderness in model 2, and measures of social capital in model 3. In the fourth model, we include all independent variables together.

Full Information Maximum Likelihood (FIML) estimation is used to deal with missing data. For continuous variables, we report fully standardized parameter estimates (expressed in terms of how many standard deviations the dependent variable changes when the predictor increases by one standard deviation). For categorical and dummy variables, we report semi-standardized parameter estimates, where only the latent variable is standardized (expressed in terms of how many standard deviations the dependent variable changes when the predictor changes from 0 to 1).

## 7 Results

Overall, the level of political cynicism among the Belgians of Turkish and Moroccan descent is quite high (see Table 1). More than half of the respondents (52.8%) agree or completely agree with the statement that there is no sense in voting because the parties do what they want anyway. Even more striking is the fact that more than 70% (completely) agree with the statements that political parties are opportunistic and that politicians are incompetent and dishonest. 63.6% of the respondents also (completely) agree with the statement about the alleged self-interest of politicians.

Table 2 displays the regression parameters of the models for political cynicism. All in all, the control variables (Model 1) explain 19.3% of the variance in political cynicism, which is quite substantial. Model 1 shows that second-generation minorities are more cynical than first-generation minorities (*β* = 0.502; *p* < 0.001). Regarding subjective income, Model 1 reveals that respondents who say that they earn more than enough (*β* = −0.863; *p* < 0.001) and just enough (*β* = −0.362; *p* < 0.01) have lower levels of political cynicism compared to those who do not earn enough to get along. Similarly, manual workers report higher levels of political cynicism than non-manual workers (*β* = 0.416; *p* < 0.001). Besides, political cynicism is not related to respondents’ city of residence, ethnic background, gender, age or religious practice.

**Table 2:** Fully and partially standardized effect parameters of model explaining political cynicism.^1^

Independent variable	Model 1	Model 2	Model 3	Model 4
**Region** = Liège (*ref. Antwerp*)	0.068	0.172	0.067	0.158
**Turkish** (*ref. Moroccan*)	0.074	0.095	0.076	0.118
**Female** (*ref. male*)	−0.042	−0.054	−0.113	−0.137
**Age** (*in years*)	0.016	0.006	0.001	−0.001
**Religious practice** *(ref. Other)*				
*Non-strictly practicing muslim*	0.148	0.018	0.163	0.048
*Strictly practicing muslim*	0.356	0.182	0.408*	0.25
**Generation** *(ref. 1st generation)*				
*2nd Generation*	0.502***	0.560***	0.496**	0.573**
*1.5 Generation*	0.295	0.332*	0.274	0.31
**Education** *(ref. Tertiary)*				
*Lower secondary*	0.175	0.199	0.143	0.158
*Higher secondary*	0.221	0.191	0.159	0.13
**Subjective income** *(ref. Not enough to get along)*				
*Just enough to get along*	−0.362**	−0.149	−0.333**	−0.099
*(More than) enough to get along*	−0.863***	−0.601***	−0.858***	−0.575***
Manual worker (*ref. No manual worker*)	0.416***	0.421***	0.414***	0.417***
**Perceived group discrimination**				
Government (*latent construct*)		0.266**		0.265***
Labor market (*latent construct*)		0.112		0.118
Daily life (*latent construct*)		0.018		0.031
**Personally experienced discrimination** *(ref. no)*		−0.039		−0.041
**Perceived ethnic outsiderness** *(ref. no)*		0.366**		0.353**
**Social capital** *(ref. No associational membership)*				
*Cross-ethnic only*			−0.089	−0.067
*Co-ethnic only*			−0.376*	−0.444**
*Both cross-and co-ethnic*			−0.261	−0.245
**Explained variance**	**0.193**	**0.285**	**0.211**	**0.308**

^1^For continuous variables, we report fully standardized parameter estimates (expressed in number of standard deviations the dependent variable changes when the predictor increases by one standard deviation). For categorical and dummy variables, we report partially standardized parameter estimates in which only the latent variable is standardized (expressed in number of standard deviations the dependent variable changes when the predictor changes from 0 to 1).

Source: Belgian ethnic minorities election study (BEMES) 2014.

In Model 2, we add the (latent) scales for perceived group discrimination (in the three domains), personally experienced discrimination and perceived ethnic outsiderness. Based on this model, no significant difference was found between respondents who reported having personally experienced discrimination in the past 5 years and those who did not. As hypothesized, perceived group discrimination turned out to be more strongly related to political cynicism, at least when considering discrimination by government agencies in particular. Perceived group discrimination by the government is positively related to political cynicism (*β* = 0.266; *p* < 0.01). As anticipated (H2), minority group members who perceive that their group is discriminated against by political institutions report higher levels of political cynicism. However, no such relationship was found for the other domains: neither perceived group discrimination on the labor market nor in daily life was significantly related to cynicism. Furthermore, respondents who were convinced that members of their ethnic group are outsiders in society reported significantly higher levels of political cynicism. Even when including perceived group discrimination, the effect size of perceived ethnic outsiderness is substantial (*β* = 0.366; *p* < 0.01), confirming hypothesis 3. Together, the predictors of group inequality – discrimination and outsiderness – add 9.2% to the explained variance in political cynicism (28.5%) compared to Model 1.

Next, Model 3 includes participation in co-ethnic or cross-ethnic associations. Remarkably, the effect parameters show that there are no significant differences in political cynicism between members of cross-ethnic associations and non-members (rejecting H4b). Interestingly, respondents who are member of associations that are composed of both cross-and co-ethnic members do not significantly differ in their political cynicism from those who are not member of any association. Yet, Moroccan and Turkish Belgians who participate exclusively in co-ethnic associations score significantly lower on political cynicism than non-members (*β* = −0.376; *p* < 0.05; confirming H4a). This suggests that bonding social capital appears to be more relevant for ethnic minorities’ attitudes towards politics than bridging capital. However, the predictive power of associational membership should not be overstated, as it only increased the explained variance of political cynicism by 1.8 percentage points (21.1%). In this sense, minorities’ involvement in associations is less predictive of political cynicism compared to perceived discrimination and outsiderness.

Including all the focal predictors simultaneously in Model 4 did not change the results substantially. The conclusions regarding our hypotheses remained the same, indicating that the relationships for the predictors of group inequality and associational membership uncovered here operate independently from one another.

## 8 Discussion

The goal of this study was to explain differences in levels of political cynicism among established ethnic minorities, in this case Belgians of Turkish and Moroccan descent. Specifically, we examined the predictive power of three mechanisms related to the intergroup context: perceived discrimination, perceived ethnic outsiderness, and social capital in the form of associational membership. Our results confirm that experiences of group discrimination by the government and of not feeling fully accepted and respected by Belgian society significantly increase political cynicism. At the same time, co-ethnic social capital has a cushioning effect on cynical attitudes.

Based on our findings, we argue that the relationship between unequal intergroup contexts and political cynicism is complex and nuanced. Our results show that personally experienced discrimination is unrelated to political cynicism, and that perceived group discrimination is relevant to political cynicism only when government agencies are the agents of discrimination. Perceived group discrimination on the labor market and in everyday life does not contribute to political cynicism. These findings support the view that political cynics within ethnic minority populations are concerned about the persistent inequalities in group status that are perpetuated by the political system. Cynics may blame political elites for the discriminatory tendencies of government agencies, but not for discrimination by employers or other citizens. Cynics are therefore less concerned with the unequal group positions *per se*. It is only when inequality is perceived as institutionalized that the position of the in-group becomes politically relevant and the responsibility of political leaders. In the cynic’s disillusionment with unequal group positions, the subject sees political and social change as impossible and comes to a ‘resigned acceptance’ (Stanley, 2007, p. 390) of the inevitability of things as they are. The rise of right-wing populist parties in Europe and their often exclusivist discourse, may be catalysts for increasing political cynicism among the established ethnic minorities, with the risk of permanent withdrawal of politics.

Furthermore, the robust relationship between perceptions of outsiderness and political cynicism indicates that political cynicism among ethnic minorities represents a mismatch between ideals of being accepted as an insider in society and the realities of exclusion and a seemingly permanent outsider position. The fact that we observe a substantial and significant relationship between ethnic outsiderness and political cynicism further suggests that political cynicism is essentially rooted in a sense of group position. The understanding that one’s ethnic group is (and always will be) an outsider in society confronts the individual with an insider establishment that is not concerned with the ethnic group’s fate in society. These circumstances are conducive to the cultivation of a more cynical worldview.

Regarding social capital, the results show that minorities who participate in organizations that are exclusively generated within the co-ethnic community are less politically cynical compared to those without membership. This is consistent with Schildkraut’s (2005) argument that in-group solidarity can protect minorities from the corrosive effects of discrimination. The observed non-significant effect of cross-ethnic social capital is somewhat surprising, but might be explained by contextual factors: Moroccan and Turkish minorities in Belgium are regularly victims of discrimination in all aspects (Alanya et al., 2017). Furthermore, participation in co-ethnic organizations may not only mitigate political cynicism but also foster a sense of community and political empowerment, contributing positively to their overall integration. This suggests that social capital, particularly within co-ethnic groups, can play a role in both reducing political cynicism and enhancing political participation. In such a context, bridging social capital might not reduce political cynicism, whereas co-ethnic organizations might act as a “‘safe haven’ from the often hostile public climate” (Maliepaard et al., 2015, p. 2635). Particularly in a context of discrimination and social exclusion, membership of co-ethnic organizations provides access to social networks and social support that are otherwise difficult for ethnic minorities to access (Maliepaard et al., 2015). In this respect, co-ethnic social capital may provide the social trust that, according to the social capital argument, ultimately reduces political cynicism.

Some shortcomings and data limitations of this study should be acknowledged. First, individual-level data do not allow to test for the effects of contextual factors, such as the degree of political representation of minority groups within the political system, prevailing policy on integration and anti-discrimination, or recent political scandals. These contextual factors can have a direct impact on citizens’ political evaluations and feelings of political efficacy (Miller and Listhaug, 1990; Marschall and Shah, 2007; Fitzgerald and Wolak, 2016; Wolak, 2017), and thus most likely also on political cynicism. Second, as with most cross-sectional studies, a key limitation of the study is endogeneity, where attitudinal indicators (perceived discrimination and ethnic outsiderness) are modeled to predict another attitudinal measure, political cynicism. Reverse causality should be considered: it could just as plausibly be argue that people who are politically cynical are more likely to feel that their group is discriminated against by the government, to retreat from civil society and to not engage in associations. At this point, the only conclusion we can draw is about the relationship between the concepts, not causality. Thirdly, the present study only looked at Moroccan and Turkish Belgians, which are two particularly visible and identifiable minority groups in the Belgian context. The results may not be representative of other, less established minority groups. We recommend that future research examines whether the effects of perceived group discrimination are similar for other ethnic minority groups. It is possible that the observed gap between personally experienced discrimination and perceived group discrimination and the observed effect of co-ethnic social capital may not be present among less identifiable and established groups. Also, as Fennema and Tillie (1999) show, the density, interconnectedness and size of the ethnic civic community is an important factor in its potential to generate social capital. Fourthly, Turkish and Moroccan minorities without Belgian citizenship or sufficient knowledge of Dutch or French did not participate in the study. Therefore, further research is needed to investigate whether the current findings can be reproduced in a sample that includes more recent migrants and first-generation migrants that do not speak Dutch or French. Likely, these ethnic minorities are less informed about Belgian politics and when asked to evaluate Belgian politicians and political institutions they may have a different comparative benchmark (the political system in the country of origin, Turkey or Morocco) resulting in more positive evaluations and less cynicism.

## Data Availability

The raw data supporting the conclusions of this article will be made available by the authors, without undue reservation.
