# Supplementary material for: The intergroup dynamics of political cynicism: how perceived discrimination, outsiderness, and social capital relate to political cynicism among Moroccan and Turkish Belgians
Source: Front Sociol. 2024 Aug 29;9:1437835. doi: 10.3389/fsoc.2024.1437835 (PMC11390568; doi:10.3389/fsoc.2024.1437835)
Supplement: Supplementary file 1 [file Data_Sheet_1.pdf]

Supplementary Material

Supplementary Figures and Tables

Table A1 Measurement equivalence of the three factors of perceived group discrimination - comparison between Belgians of Turkish and Moroccan descent.

Table A2. CFA Results for Perceived Group Discrimination

Table A3. Descriptive Statistics

Table A1. Measurement equivalence of the three factors of perceived group discrimination – comparison between Belgians of Turkish and Moroccan descent.

	Chisq	Df	Chisq diff	Df diff	Pr(>Chisq)	RMSEA	CFI	TLI
Configural equivalence	144.25	64				0.052	0.968	0.955
Metric equivalence	149.06	71	4.3831	7	0.7347	0.048	0.969	0.961
Scalar equivalence	156.59	78	7.5457	7	0.3743	0.046	0.969	0.964

Table A2. CFA Results for Perceived Group Discrimination

	% Agree + Completely Agree	Perceived Group Discrimination		
		Government	*Labor market*	*Daily Life*
Standardized Factor Loadings				
If we need something from the government, people like me have to wait longer than others.	36.4	.554		
Government does more for Belgians than for people of my descent	24.6	.677		
Social and city services view people of my descent as a burden	33.8	.645		
It is more difficult for people of foreign descent to get access to social services	26.4	.587		
It is more difficult to find a job for people of a foreign descent.	75.1		.703	
People of [Moroccan/Turkish] descent only find work below their educational level.	41.8		.727	
To find a steady job in Belgium is very difficult for people of a foreign descent.	55.7		.790	
How often does it occur according to you that people of [Turkish/Moroccan] descent in Belgium experience hostility or unfair treatment because of their descent or background...	% Regularly + Often			
At school or at the workplace.	20.6			.781
Going out, in dancings, cafés or restaurants.	23.6			.677
On the street or in public transport.	15.0			.733
Correlations between factors				
Perceived Group Discrimination: Government				

Perceived Group Discrimination: Labor Market	.645		
Perceived Group Discrimination: Daily Life	.141	.174	

3-factor model fit: *Chi-square:102.112; df: 32; RMSEA: .050; CFI: .969; TLI: .956*

1-factor model fit: Chi-square: 946.321; df: 35; RMSEA: .173; CFI: .598; TLI: .483

Source: Belgian Ethnic Minorities Election Study (BEMES) 2014

**Table A3.** Descriptive Statistics	
	BEMES 2014
**Political Cynicism**	Mean (SD) / %
There's no sense in voting; the parties do what they want to do anyway.	3.36 (1.14)[1]
Parties are only interested in my vote, not in my opinion.	3.79 (0.93)
Most politicians promise a lot, but don't do anything.	3.80 (0.92)
As soon as they are elected, politicians think they are better than people like me.	3.66 (0.99)
**Perceived Group Discrimination: Government**	
If we need something from the government, people like me have to wait longer than others. (1-5)	2.95 (1.05)
Government does more for Belgians than for people of my descent.	3.43 (0.88)
Social and city services view people of my descent as a burden.	2.98 (0.98)
More difficult for people of foreign descent to get access to social services.	2.75 (0.97)
**Perceived Group Discrimination: Labor Market**	
It is more difficult to find a job for people of a foreign descent.	3.81 (0.96)
People of [Moroccan/Turkish] descent only find work below their educational level.	3.15 (1.07)
Finding a steady job in Belgium is very difficult for people of a foreign descent.	3.45 (1.06)
**Perceived Group Discrimination: Daily Life**	
Perceived discrimination at school or at the workplace.	2.61 (1.18)
Going out, in dancings, cafés or restaurants.	2.88 (1.23)
On the street or in public transport.	2.54 (1.31)
**Personally Experienced Discrimination: During last 5 years**	
Rarely - Often	48.05%
Never	51.95%
**Perceived Ethnic Outsiderness**	
"A person of Turkish/Moroccan descent can never fully participate in Belgian society – no matter how hard they try" (ref.)	18.15%
"A person of Turkish/Moroccan descent can participate fully in Belgian society"	81.85%

[1] All numeric variables (except for age) are measured as 5-point scales.

**Associational membership**	
No Membership (ref.)	61.96%
Cross-Ethnic only	17.00%
Co-Ethnic only	11.99%
Both co-ethnic and cross-ethnic	9.55%
**Highest obtained level of education**	
Lower Secondary	35.54%
Higher Secondary	47.27%
Tertiary (ref.)	16.97%
**Subjective Income**	
“We do not have enough” (ref.)	20.34%
“We have just enough to make ends meet”	43.10%
“We have enough to make ends meet without difficulties”	30.46%
“We have more than enough, we can easily save money”	6.09%
**Labor Status**	
No Manual Worker (ref.)	62.41%
Manual Worker	37.59%
**Generation**	
2nd Generation	62.64%
1.5 Generation	20.62%
1st Generation (ref.)	16.74%
**Age (in years)**	32.52 (11.33)
**Gender**	
Male (ref.)	49.09%
Female	50.91%
**Religious Practice**	
Strictly Practicing Muslim	47.42%
Non-Strictly Practicing Muslim	40.37%
Other (ref.)	12.10%
